# pyInfinityFlow: optimized imputation and analysis of high-dimensional flow cytometry data for millions of cells

**DOI:** 10.1093/bioinformatics/btad287

**Published:** 2023-04-25

**Authors:** Kyle Ferchen, Nathan Salomonis, H Leighton Grimes

**Affiliations:** Cancer and Cellular Biology, University of Cincinnati, Cincinnati, OH 45229, United States; Immunobiology, Cincinnati Children’s Hospital Medical Center, Cincinnati, OH 45229, United States; Biomedical Informatics, Cincinnati Children’s Hospital Medical Center, Cincinnati, OH 45229, United States; Department of Pediatrics, University of Cincinnati, Cincinnati, OH 45229, United States; Immunobiology, Cincinnati Children’s Hospital Medical Center, Cincinnati, OH 45229, United States; Department of Pediatrics, University of Cincinnati, Cincinnati, OH 45229, United States; Experimental Hematology and Cancer Biology, Cincinnati Children’s Hospital Medical Center, Cincinnati, OH 45229, United States

## Abstract

**Motivation:**

While conventional flow cytometry is limited to dozens of markers, new experimental and computational strategies, such as Infinity Flow, allow for the generation and imputation of hundreds of cell surface protein markers in millions of cells. Here, we describe an end-to-end analysis workflow for Infinity Flow data in Python.

**Results:**

pyInfinityFlow enables the efficient analysis of millions of cells, without down-sampling, through direct integration with well-established Python packages for single-cell genomics analysis. pyInfinityFlow accurately identifies both common and extremely rare cell populations which are challenging to define from single-cell genomics studies alone. We demonstrate that this workflow can nominate novel markers to design new flow cytometry gating strategies for predicted cell populations. pyInfinityFlow can be extended to diverse cell discovery analyses with flexibility to adapt to diverse Infinity Flow experimental designs.

**Availability and implementation:**

pyInfinityFlow is freely available in GitHub (https://github.com/KyleFerchen/pyInfinityFlow) and on PyPI (https://pypi.org/project/pyInfinityFlow/). Package documentation with tutorials on a test dataset is available by Read the Docs (pyinfinityflow.readthedocs.io). The scripts and data for reproducing the results are available at https://github.com/KyleFerchen/pyInfinityFlow/tree/main/analysis_scripts, along with the raw flow cytometry input data.

## 1 Introduction

Flow cytometry enables single-cell proteomic profiling of millions of cells, using fluorescent-conjugated antibodies to extracellular or intracellular proteins. The number of molecules that can be profiled using antibody-based strategies has increased with the development of new technologies, such as novel fluorescent proteins, spectral deconvolution (Liechti et al. 2021), heavy-metal-conjugated antibodies detected with mass spectrometry (Bandura et al. 2009), and oligo-nucleotide-conjugated antibodies detected with next-generation sequencing (Stoeckius et al. 2017). While approaches such as fluorescence-based Flow Cytometry Activated Cell Sorting (FACS) provide the means to effectively enrich for cell populations, they are limited by the number of simultaneously measurable fluorophores. Thus, to develop reliable flow cytometry panels to detect rare cell populations for diverse lineages, methods are required to simultaneously measure potentially hundreds of markers across millions of cells.

Infinity Flow was originally developed as a low cost and efficient experimental protocol to scale conventional flow cytometry from dozens to hundreds of markers, without increasing the repertoire of simultaneously measured spectra (Dutertre et al. 2019). The associated R package computational workflow, called infinityFlow, is able to integrate markers (infinity markers) measured in individual flow cytometry captures, as long as each capture shares a common set of backbone markers (Becht et al. 2021). An optimal backbone panel consists of a sufficiently diverse and informative set of antibodies that span all major evaluated cell lineages. Cells are stained with backbone antibodies, split into individual wells and stained for the additional query target. The query antibodies share a fluorochrome [e.g. phycoerythrin (PE)]. Using nonlinear regression with machine learning, infinityFlow imputes the expression of exploratory Infinity markers in individual Flow Cytometry Stand (FCS) files (sharing the backbone markers) to the pool. With this strategy, imputed flow cytometry datasets can span a diversity of features to distinguish broad and subtle cell population differences, comparable to high-throughput genomic assays. Thus, Infinity Flow allows for the integration of hundreds of separately profiled markers. While powerful, the existing R package infinityFlow workflow cannot be readily applied to millions of cells and hundreds of imputed markers (down-sampling required), is not flexible to new types of Infinity Flow study designs, has significant memory requirements, and does not directly interface with downstream supervised or unsupervised clustering approaches.

To improve upon and expand the original workflow, we introduce pyInfinityFlow, a Python implementation of infinityFlow with improved efficiency in speed and memory, and support for advanced unsupervised and supervised anndata-dependent single-cell analyses (Scanpy, pytometry, MarkerFinder, cellHarmony) (Wolf et al. 2018; DePasquale et al. 2019; Büttner et al. 2022). pyInfinityFlow is more flexible to the options applied, and is extensible to more complex study designs that include multiple Infinity markers per panel (see Supplementary Information).

## 2 Design

Similar to the original R package infinityFlow workflow, pyInfinityFlow was developed to impute new surface markers into an existing flow cytometry backbone panel using XGBoost regression. XGBoost (Chen and Guestrin 2016) has been well-demonstrated to provide optimal balance of performance time and prediction accuracy (Becht et al. 2021). The predictors of the model are referred to as “Backbone markers” and the response variable is referred to as an “Infinity marker.” The pipeline processes one or more sets of Infinity marker FCS files to train the regression model. The model is then used to predict the Infinity marker signal on either a single FCS reference file or a pool of events from the Infinity marker FCS files to build a final dataset with all features (held in memory as an AnnData object). Each FCS file records the light emitted after an event of a cell, multiplet, or debris moving over the detectors is recorded in multiple channels. Isotype background correction can optionally be used in pyInfinityFlow and was re-implemented with a linear model as previously described (Supplemental Methods) (Becht et al. 2021).

**Figure 1.**
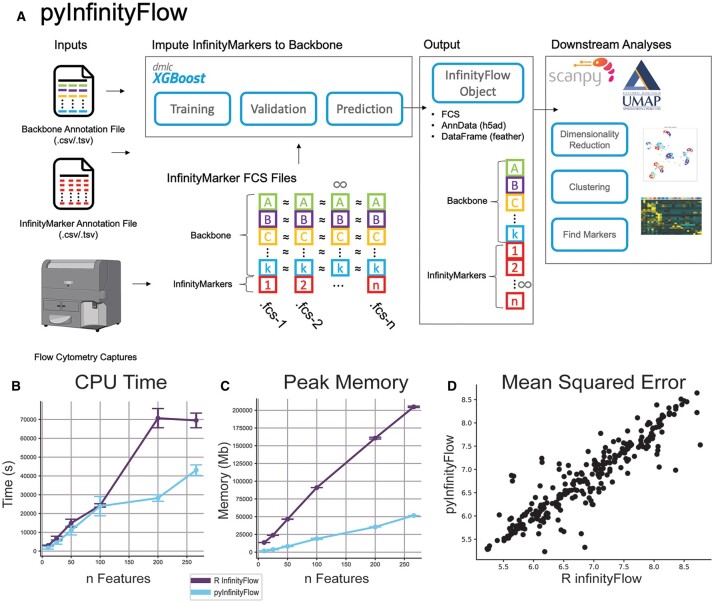
pyInfinityFlow: overview of features and performance. (A) Graphical abstract of pyInfinityFlow package. (B–D) Comparison of pyInfinityFlow and R infinityFlow packages using the mouse lung dataset with varying numbers of Infinity markers. pyInfinityFlow required a lower CPU time (B), lower Peak Memory (C), and had similar accuracy in regression as measured by mean squared error on a log10 scale (D).

In addition to these options, pyInfinityFlow includes additional options for data scaling and normalization; flexibility for more complex study designs; and downstream analyses for dimensionality reduction, clustering, and cell-type marker identification (Fig. 1A). These include both new interfaces (MarkerFinder, cellHarmony) and calls to existing interfaces where possible (Supplemental Methods). Specifically, the user can pool events from the Infinity marker FCS files or supply a separate FCS file to serve as the Backbone data for the final Infinity Flow object. The workflow can be run as a full pipeline (single command) or as independent steps, making it usable by both data scientists and biologists. Alternative normalization and correction strategies are introduced to improve data imputation. These include Isotype background correction and logicle normalization. The final Infinity Flow object can be stored as sparse data objects (h5ad) or as a data frame stored in a binary feather file format, enabling direct manipulation with Scanpy, or other tools, to identify broad and rare cell populations with Leiden clustering (Traag et al. 2019) and run UMAP dimensionality reduction on millions of cells (McInnes et al. 2018). The MarkerFinder algorithm (Venkatasubramanian et al. 2020) is used in pyInfinityFlow to identify unique distinguishing cell surface markers for Leiden-defined cell populations. Finally, this package provides both an API for fine tuning of the analysis pipeline parameters as well as command line utilities for simple execution. The API is split into four modules (fcsio, Transformations, Plotting Utilities, and Debugging Utilities) that handle different aspects of processing Flow Cytometry data with Python.

## 3 Performance

To assess the performance of pyInfinityFlow, we analyzed a prior mouse lung Infinity Flow dataset using the same parameters as previously described (Becht et al. 2021) (Supplementary Information). For each Infinity marker, an FCS file was captured with at least 20 000 events. These events are first split into training and validation sets by randomly sampling 50% for training and using the remaining 50% for validation. Thus, for each individual capture, half of the cells were used to train the XGBoost regression model and the other half were used to compute the validation metrics. To build the final dataset, 10 000 events from the validation sets are randomly selected and concatenated into a single expression matrix. We find that pyInfinityFlow outperforms the R implementation in terms of computational speed and memory efficiency with increasing panel size (Fig. 1B and C). The increased performance does not impact the regression accuracy, as measured by mean absolute error (Fig. 1D). We were able to observe similar performance improvements with pyInfinityFlow for increasing cell numbers relative to the R version (Supplementary Fig. S1). pyInfinityFlow is compatible with datasets of over 332 imputed markers (Dutertre et al. 2019) and >3 million input cells for imputation, without down-sampling (32 504 s with 40 GB of RAM). In contrast, the R implementation encountered a memory error when given 800 GB of RAM. The calculation of the MSE values to compare accuracy between the R-package infinityFlow and pyInfinityFlow was done on the 10 000 events that are pooled into the final Infinity Flow object. This ensures that MSE values are not biased by changes in the number of input cells. The output of pyInfinityFlow consists of the concatenated backbone channels, imputed Infinity marker channels, and original channels not imputed and not part of the backbone. This single-cell dataset, stored as an AnnData object in memory, was dimensionally reduced and clustered using wrapper functions in pyInfinityFlow to Scanpy methods for principal component analysis (PCA), UMAP, and Leiden clustering. The embedded pyInfinityFlow MarkerFinder implementation was then used to identify markers for these Leiden clusters.

*Z*-score normalization has been noted as a necessary feature of the R-package infinityFlow method but has not been previously assessed to determine its requirement. To assess the necessity of *z*-score normalization, we profiled over 2 million murine hematopoietic progenitors with 21 backbone markers with Infinity Flow. We selected 11 out of the 21 backbone markers as a surrogate backbone to evaluate prediction of the remaining 10. Using the established ground-state truth, we find that *z*-score normalization (designed to reduce batch effects between samples), introduces significant artifacts in the regression predictions (Supplementary Information, Supplementary Fig. S2). Hence, while *z*-score normalization is included in the method, by default, it is not applied.

To illustrate the utility of high-dimensional datasets generated by pyInfinityFlow, we mapped scRNA-seq defined populations from the mouse lung cell atlas to the surface protein expression using an embedded call to the cellHarmony (DePasquale et al. 2019) module of pyInfinityFlow. The gene names encoding the surface proteins defined in the pyInfinityFlow dataset were used to rename the protein feature labels to provide links between the scRNA-seq and Infinity Flow dataset features. We find that the cellHarmony mapping of labels to the pyInfinityFlow dataset identifies challenging-to-detect rare lung cell populations that can be resolved through sub-clustering of tens of thousands of cells (Supplementary Fig. S3, Supplementary Text). While the mapping RNA to antibody profiles has been shown to be highly inferential (Triana et al. 2021; Dou et al. 2022), lineage predictions broadly matched to those made by the original authors, while highlighting potential novel cellular subsets (Becht et al. 2021). These novel populations included highly similar mesenchymal alveolar fibroblasts (AF1, AF2), smooth muscle (VSMC, ASMC), pericytes, and secondary crest myofibroblasts (SCMF). To determine whether pyInfinityFlow could resolve these rare predicted subtypes, we further sub-clustered our initial predicted fibroblasts cluster, to define multiple new sub-clusters (Supplementary Fig. S4A and B). This analysis confirms the presence of fibroblast and smooth muscle cell populations that could previously only be resolved through extensive single-cell genomics and functional studies and nominates new cell-surface markers for their isolation (Guo et al. 2022) (Supplementary Fig. S4C–E). Such analyses open the door for gating and isolating these other rare cell populations, with a novel repertoire of distinguishing marker antibodies (Supplementary Figs S3 and S4).

## 4 Conclusions

pyInfinityFlow represents an optimal workflow for the analysis of millions of flow cytometry profiles in Python, significantly extending the repertoire of analyses of the original implementation. This allows users to build datasets with larger numbers of cells, which is necessary to profile rare and transitional cell populations. To this point, we have shown that pyInfinityFlow in combination with label transfer using cellHarmony can be used to identify rare populations that were not previously found in scRNA-seq from mouse lung (e.g. SVEC, respiratory airway secretory cells). Additionally, the user has more flexibility to specify a separate FCS reference file, to use the pipeline without isotype controls, to generate AnnData structured outputs, to cluster the flow cytometry data, and to find markers for populations. The AnnData data structure allows for the quick and simple application of single-cell analysis tools built into Scanpy, such as dimensionality reduction and clustering. Extensions of Scanpy such as pytometry, a package built for analyzing cytometry data, can also be quickly applied to datasets generated using pyInfinityFlow (Büttner et al. 2022). The package provides both a command line tool, enabling wide adoption by users without programming experience, as well as an API to provide precise control of analysis parameters. While approaches for normalization were shown to be more deleterious than advantageous, it will be necessary to assess alternative approaches to further optimize the imputation of antibody signals in extremely large datasets. Looking forward, we hope to develop extensions to this approach to improve the identification of rare cell populations similarly reflected in emerging single-cell genomics studies.

## Supplementary Material

btad287_Supplementary_Data
