# Supplementary material for: pyInfinityFlow: optimized imputation and analysis of high-dimensional flow cytometry data for millions of cells
Source: Bioinformatics. 2023 Apr 25;39(5):btad287. doi: 10.1093/bioinformatics/btad287 (PMC10166583; doi:10.1093/bioinformatics/btad287)
Supplement: btad287_Supplementary_Data [file btad287_supplementary_data.zip › supplementary_text.pdf]

Supplementary Information

Background on Infinity Flow

The purpose of Infinity Flow is to use regression to expand the number of potential fluorescent markers that can be used to describe a population by flow cytometry (Becht, et al., 2021; Dutertre, et al., 2019). It requires running multiple samples from the same population of interest with overlapping fluorescent markers in each panel, referred to as the backbone. The backbone markers serve as the independent variables of the regression model to predict the signal of the remaining query marker(s) (referred to as Infinity markers), or the dependent variables in the regression model. The regression models can then be used to impute the signals of the Infinity Markers onto any sample of cells from the same population that has been stained with the set of backbone markers. This differs from traditional flow cytometry in which a fixed number of markers in a panel is applied once to a sample.

Infinity Flow was originally applied to screening kits in which hundreds of PE-conjugated antibodies are individually aliquoted into multiple 96-well plates. A backbone panel of antibodies was used to stain a large sample of cells, then aliquoted to the wells of the 96-well plates to stain for the PE-conjugated Infinity markers. Flow cytometric analyses of the cells in these wells results in hundreds of panels, each of which is stained for the backbone set of markers in addition to the varying PE-conjugated Infinity marker.

However, the approach is not theoretically limited to this configuration, in which a single Infinity marker is predicted with each panel. For example, a more complex configuration could involve having multiple Infinity markers in one of the individual panels. pyInfinityFlow adds support for these more complicated configurations by allowing the user to annotate which channels for each input FCS file should be used for backbone markers in the "backbone annotation file" and which channels should be treated as dependent variables for the regression model in the "Infinity marker annotation file".

Overall Software Design

The pyInfinityFlow package was designed to facilitate the integrative analysis and imputation of multi-sample Flow Cytometry data in Python. This implementation consists of command line tools that require no Python scripting (single-command option) in addition to a well-documented API that lets users significantly parameterize the pipeline.

The Infinity Flow experimental analysis pipeline starts with the construction of a backbone panel. This is the set of diverse pan-lineage markers used as the predictors in the

regression model. The backbone panel is combined with an Infinity Marker (variable marker that differs for each flow sort) to build a regression model that allows for the prediction of the Infinity Marker signal given the expression of the backbone markers. This enables the imputation of all infinity marker signals across all cells and all independent flow cytometry sorts. The final Infinity Flow object refers to the set of cells on which the backbone features, imputed Infinity Markers, and additional FCS parameters not used in the regression steps that are merged into a single dataset.

As compared to the original R implementation, pyInfinityFlow provides greater flexibility with substantially improved runtime and greater memory efficiency. Some steps in the original R implementation are included but are now optional, as they can negatively affect performance. For example, a major application of Infinity Flow is for the identification and subsequent isolation of cell populations using Fluorescence-Activated Cell Sorting (FACS). However, conventional FACS cannot consider background correction requiring that background correction. Hence, methods for isotype control consideration are optional. pyInfinityFlow further adds additional flexibility to supplied experimental design. For example, users can provide a separate FCS reference file to use as the final dataset, as opposed to pooling of cells from each of the individual Infinity Markers. If a user has markers that are not well explained by the backbone, providing a separate FCS reference file with that marker ensures accurate representation of that signal. The logicle normalization step is also optional. While for most fluorescent signals, logicle normalization represents a reliable and important approach to remove the effects of outliers, there may be instances where the user wishes to supply a custom data normalization strategy. Conversely, as we demonstrate below, other normalization strategies, such as z-score normalization of the backbone can introduce unintended artifacts that negatively impact the integration of data across multiple FCS files.

Methods

The pyInfinityFlow package extends the functionality of anndata Python objects to provide additional analysis features that are not present in the original R implementation. The command line tool provides the option to cluster the final Infinity Flow object using the Leiden clustering algorithm implemented in Scanpy. The API tutorial provided in the documentation walks users through using this function in a Python script. The API is divided into four principal modules (fcsio, Transformations, Plotting Utilities, Debugging Utilities). This modular design allows for analyses to be partially completed and run, without re-performing individual steps (intermediate outputs stored frozen binaries or h5ad). For imputation of infinity markers, the XGBoost python

library is used to compute a non-linear regression model, due to its ability to scale well with large datasets and prior reported accuracy (Becht, et al., 2021). First, the feature space is reduced using PCA to work with large cell numbers. A nearest neighbor graph is generated for the data prior to carrying out UMAP and Leiden clustering. Both methods use the nearest neighbor graph to visualize the cells and group them into communities. UMAP was selected as it better preserves global lineage relationships over alternatives. Parallel processing is facilitated through built-in methods within XGBoost and in the UMAP python library. Optional normalization approaches include Logicle normalization (Moore and Parks, 2012), which is a standard normalization approach for flow cytometry data in which the data is re-scaled to a range of 0-1, z-score normalization on the backbone markers to reduce variability between FCS files and isotype control signal correction to correct for non-specific antibody binding (linear regression). Unlike the original implementation, isotype control normalization is not required in pyInfinityFlow. These algorithms were re-implemented in Python using statistical libraries (scikit-learn). To identify optimal markers, we reimplemented the python 2.7 algorithm MarkerFinder (AltAnalyze software). This method efficiently evaluates the specificity of a given marker to a single-cell cluster of interest versus all other cells (two groups) (Venkatasubramanian, et al., 2020).

Machine Learning Approach

The XGBoost algorithm is used in the pyInfinityFlow package to impute the fluorescence signal of one channel in a captured panel to another by using overlapping channels as predictors. This enables hundreds of fluorescence channels to be imputed together if each shares the same predictor channels. For every predicted channel, an instance of an XGBoost model is created and stored in the CombinedRegressionModels object in pyInfinityFlow. The training and testing datasets are created by splitting the events of the input FCS file into mutually exclusive sets. By default, 80% of the events of the input FCS file are used for training and the remaining 20% are used for testing. The XGBoost model is trained on the training set and tested independently with the testing dataset. The performance is measured using mean squared error. After the testing step, pyInfinityFlow adds an error_metrics attribute to the CombinedRegressionModels object to store the performance metrics. To assess the error introduced by normalization, we used separate biological replicates for training and testing datasets.

Software, Analysis Code, and Data Accessibility

All the software for pyInfinityFlow, analysis scripts, test dataset, and documentation is available on a GitHub repository (https://github.com/KyleFerchen/pyInfinityFlow). The package is also

stored in the Python Packaging Index (https://pypi.org/project/pyInfinityFlow/). The documentation for the package is hosted on Read the Docs and covers instructions for installation, tutorials for the command line tools, the full API documentation, and a tutorial to walk through using the API in a complete data analysis pipeline (https://pyinfinityflow.readthedocs.io/en/latest/). The mouse lung dataset was previously made available on the Flow Repository website (https://flowrepository.org/id/FR-FCM-Z2LP). A subsample of this dataset consisting of the first 10 InfinityMarkers and their isotype control antibodies was saved in the pyInfinityFlow GitHub repository (https://github.com/KyleFerchen/pyInfinityFlow/tree/main/example_data). The Kit+ bone marrow dataset was made publicly available on the Flow Repository website (https://flowrepository.org/id/FR-FCM-Z5UK).

Code Implementations

Where possible, pyInfinityFlow uses previously written wrappers- or existing- implementations of functions from other Python packages. Functions that are frequently used for scRNA-seq data analysis are provided by Scanpy, which act on anndata objects. Implementations that are specific to pyInfinityFlow were written to either speed up existing implementations or to provide additional functionality. For example, pyInfinityFlow has its own implementation of a class to handle the reading and writing of FCS files so that it can quickly identify the essential elements of the file and read only what is needed. Here is a list of the major functionalities provided by pyInfinityFlow, and how they are implemented:

File Input/Output

- FCS files: custom implementation "fcs_io" module
- Annotation files: pandas package
- Binary feather file output: pandas package
- H5AD output: anndata

Scaling

- For plotting marker plots: wrapper for sklearn.preprocessing.MinMaxScaler

Normalization

- Logicle: custom implementation in "Transformations" module
- Z-score: wrapper for scipy.statas.zscore

Dimensionality Reduction

- PCA: wrapper to Scanpy's wrapper to scikit-learn
- UMAP: wrapper to Scanpy's wrapper to UMAP package

Clustering

- Leiden clustering: wrapper to Scanpy's Leiden Clustering method

Cell-type marker identification

- MarkerFinder: custom Python3 re-implementation of existing function in AltAnalzye package (Python2)

Regression

- Infinity Markers: Wrapper to XGBoost package implementation
- Linear model for isotype background correction: wrapper to sklearn package

Visualizations

- Scatterplots: implemented using matplotlib
- Heatmaps: implemented using matplotlib

Data Preparation and Inputs

pyInfinityFlow requires four standard inputs: 1) FCS files, 2) output path, 3) backbone annotations and 4) infinity marker annotations. For input 1, the FCS files that capture the backbone panel with a given Infinity Marker must be saved in a directory, and this directory must be provided. For input 2, the user must specify an output directory in which to save each of the outputs. For input 3, the user must supply a backbone annotation file which specifies for each backbone marker the following features: (1) the channel name in the reference FCS file, (2) the channel name in the query Infinity Marker FCS file, (3) the final name to assign to the channel in the output. If the user is pooling events from each Infinity Marker FCS file into the final Infinity Flow, then the reference and query channel name will be identical. However, if a separate reference FCS file is given, we recognize there may be a difference in the channel name, so we allow the user to account for this difference in this input. The fourth and final input the user needs to prepare is an Infinity Marker annotation file. This annotation file specifies, for each of the Infinity Markers (targets of the regression model, which are the signals we need to impute): (1) the name of the FCS file as it exists in the input directory, (2) the channel name that needs to be imputed with the regression model, (3) the name to give to the final channel (note that channel names need to be unique in an FCS file), and (4) the name of the isotype control to use for background correction (this fourth column is optional). It is possible for multiple Infinity Markers to be captured in the same FCS file. The user simply needs to have a row in this file for each Infinity Marker.

Running the Software

With these inputs, pyInfinityFlow imports the data, performs normalization (optional), and trains a regression model. FCS files are loaded into memory in Python as anndata objects, with specific details on how Logicle normalization should be performed designated in the var attribute. The pipeline will first create the FileHandler object in Python, which is a way to store, for each InfinityMarker, the file from which to read the data, the FCS directory, which events in the file to use for training, validation, and pooling (if a separate FCS reference is not used). The user has the option to normalize fluorescence channels with Logicle normalization. This is a way to put the data on a linear-like scale to reduce the effect of outliers. It will not be applied to channels that are normally represented on a linear scale, like forward or side scatter. The user also has the option to normalize the backbone channels with a z-score transformation. This was originally implemented in the original R package to minimize the batch effects between captures. After the data is loaded and normalized, an XGBoost regression model is trained to predict the level of each Infinity Marker. The default parameters are used for this model unless the user provides a dictionary with keyword arguments to the pyInfinityFlow API function.

After training a model for each Infinity Marker, the XGBoost "predict" function is used to impute the signal on a reference dataset. The user can either pool events from the Infinity Marker FCS files or provide a separate FCS file to use as the reference. The same normalization methods used prior to model fitting should be used prior to this prediction step. The final Infinity Flow object is then stored in memory as an anndata object, and consists of (1) the backbone channels, (2) the imputed Infinity Marker channels, and (3) the additional features from the reference FCS file(s) not used in any regression models.

With the Infinity flow object as an anndata object, the user can apply the same functions used in the Scanpy as for single-cell RNA sequencing analyses. The pipeline will specifically use PCA, UMAP, and Leiden clustering as optional downstream analyses. MarkerFinder assigns features to their most enriched Leiden clusters. It calculates the Pearson correlation coefficient between the expression values of each marker to an idealized vector in which each cell of a given cluster is assigned a value of 1, and all other cells are assigned a value of 0. A MarkerFinder p-value is calculated using a t-statistic calculated from the MarkerFinder Pearson correlation coefficient. The Pearson correlation coefficient is calculated using matrix multiplication to parallelize each marker to group comparison as efficiently as possible. Optionally, the backbone, Infinity Marker features, and Leiden clusters are plotted over the UMAP coordinates and saved as files. The MarkerFinder results are stored as a table as well as a heatmap with the top 50 cells from each cluster as columns and their zscore normalized

expression value of each feature as rows. Two tables are provided: (1) each marker is assigned to one population with its highest Pearson correlation coefficient value, and (2) the top 5 markers for each cluster are written. The user can save the InfinityFlow object as an H5AD file (preserving the anndata structure), an FCS file (to use with other Flow Cytometry analysis tools like FlowJo), or a feather file (dataframe to quickly load into Python).

To compare the performance of pyInfinityFlow to the R infinityFlow package, the previously published and reposited mouse lung data set was used (Becht, et al., 2021). This dataset consists of a 14-color backbone (CD4, CD44, CD8a, CD11c, CD11b, F480, Ly6C, Lineage, CD45, CD103, CD24, CD69, CD301b, MHCII, Live/Dead) with 252 separate PE-conjugated antibodies each matched to 1 of 14 isotype controls to use for background correction. The data was processed in the same way as the original publication, using half of the cells present in each InfinityMarker FCS file for fitting an XGBoost regression model, and the remaining half of the cells for validation. The final Infinity Flow object is created by pooling 10,000 events from the validation set and using the regression models to impute each InfinityMarker based on the backbone panel. We tested varying numbers of InfinityMarkers (10, 25, 50, 100, 200, and 252) and assessed the computation time, peak and average memory usage, and the mean squared error metric of the regression predictions. We also saved the time it took for various regression steps in the pipeline. All these tests were performed on a CentOS Linux based HPC cluster at Cincinnati Children's Hospital Medical center as a bsub job with 250 GB memory and 12 cores.

In addition to varying the numbers of markers used in imputation, we also tested varying the number of input cells and calculated the run time of multiple processes in the Infinity Flow imputation steps. A subset of 10 random markers from the mouse lung dataset was selected and the Infinity Flow analysis pipeline was carried out using increasing cell number input sizes (equal for both fitting and prediction steps). The runtime for file reading, logicle normalization, z-score calculation, XGBoost regression model fitting, prediction and validation measurements, and linear model background correction were calculated for each input cell sizes. We find the pyInfinityFlow implementation scales better with increasing cell numbers, illustrating its improved performance (**Supplemental Figure 1**).

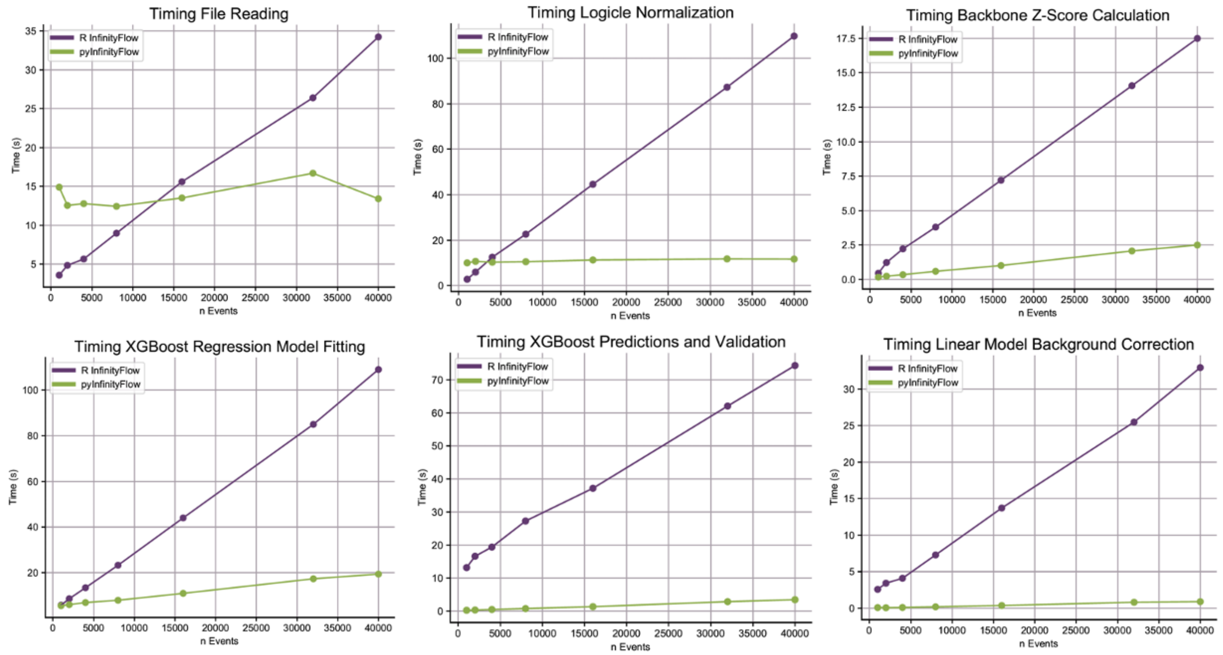

Supplementary Figure 1. Improved performance scaling with increasing cell numbers. Line charts showing the computation time for various processes of the imputation steps for both pyInfinityFlow (green) and R infinityFlow (purple).

To compare the performance on a dataset with more markers, we carried out the analysis on data collected from peripheral blood mononuclear cells (PBMCs) stained using the Biolegend LegendScreen Human PE kit from the first use of the Infinity Flow protocol (Dutertre, et al., 2019). This article preceded the development of the R infinityFlow package. We compared the performance of pyInfinityFlow to R infinityFlow by applying the following analysis settings: 50% of the cells were randomly selected and used from each LegendScreen marker stain to train the XGBoost regression model and the remaining 50% from each was used for validation and pooling. Up to 10,000 events were pooled from each file from the validation set to build the final Infinity Flow object. Not all files had up to 20,000 events total, in which case all available events (<10,000) from the validation set of that file were included. pyInfinityFlow was able to complete the construction of the Infinity Flow object with a CPU time of 32,504 seconds and a peak memory of 40 GB. The R infinityFlow instance encountered a memory error, requesting more RAM than could be provided by our computing cluster (>800 GB). This illustrates the importance of memory efficiency when working with larger datasets.

Isotype Background Correction

Isotype background correction is used to try to remove background fluorescence from the imputed Infinity Marker signal. It is known that fluorescently conjugated antibodies can bind non-specifically, through mechanisms that are not related to the target epitope. For example, it has previously been demonstrated that Fc-receptors can non-specifically bind fluorescently conjugated antibodies (Andersen, et al., 2016) and that this effect could be mitigated by staining the cell sample with Fc-blocking antibodies prior to staining with fluorescently conjugated antibody panel. In addition, it is possible that part of the background fluorescence signal is caused by non-specific binding through interactions of parts of the antibody specific to the class and host species in which the antibody was developed. Becht et al. implemented a method to try to correct for the effects of the class and host species of the fluorescently conjugated antibody. Each Infinity Marker is matched to an isotype control Infinity Marker by class and host species and a regression model is built for both to predict its signal for each output cell. To perform background correction, a linear model is built in R using the "lm" function with the isotype background Infinity Marker as the independent variable and the target Infinity Marker as the dependent variable. The corrected signal is the residual of the linear model for each cell.

In pyInfinityFlow, the same procedure is used to correct for background fluorescence associated with the species and class of the isotype control Infinity Marker. The "perform_background_correction" function in the " InfinityFlow_Utilities" module acts as a wrapper for the "LinearRegression" class from "sklearn.linear_model". The user is meant to specify the isotype control Infinity Markers used for background correction in the Infinity Marker annotation file (csv/tsv file), which is either an input to the command line tool or read into a pandas dataframe through the "read_annotation_table" function in the "InfinityFlow_Utilities" module. Each isotype control should be an Infinity Marker row in this annotation file (see the table below, outlined in red). The user specifies which isotype Infinity Marker to use for correcting the background for each Infinity Marker (outlined in blue). The user can choose not to carry out background correction by omitting the fourth column of this file. This is different from the R implementation of Infinity Flow, which requires an isotype control Infinity Marker for each Infinity Marker for the program to run.

File	Channel	Name	Isotype
backbone_Plate2_G1_G01_073_target_33D1.fcs	FJComp-PE(yg)-A	33D1	Isotype_rIgG2b
backbone_Plate2_F7_F07_067_target_Allergin-1.fcs	FJComp-PE(yg)-A	Allergin-1	Isotype_mIgG1
backbone_Plate2_F8_F08_068_target_B7-H4.fcs	FJComp-PE(yg)-A	B7-H4	Isotype_AHIgG
backbone_Plate1_A2_A02_002_target_CD1.fcs	FJComp-PE(yg)-A	CD1d	Isotype_rIgG2b
backbone_Plate1_G4_G04_076_target_CD103.fcs	FJComp-PE(yg)-A	CD103	Isotype_AHIgG

backbone_Plate1_G5_G05_077_target_CD105.fcs	FJComp-PE(yg)-A	CD105	Isotype_rIgG2a
backbone_Plate1_G6_G06_078_target_CD106.fcs	FJComp-PE(yg)-A	CD106	Isotype_rIgG2a
backbone_Plate1_G7_G07_079_target_CD107a (Lamp-1).fcs	FJComp-PE(yg)-A	CD107a (Lamp-1)	Isotype_rIgG2a
backbone_Plate1_G8_G08_080_target_CD107b (Mac-3).fcs	FJComp-PE(yg)-A	CD107b (Mac-3)	Isotype_rIgG1
backbone_Plate1_G9_G09_081_target_CD115.fcs	FJComp-PE(yg)-A	CD115	Isotype_rIgG2a
backbone_Plate3_F12_F12_072_target_Isotype_rIgG2b.fcs	FJComp-PE(yg)-A	Isotype_rIgG2b	Isotype_rIgG2b
backbone_Plate3_F6_F06_066_target_Isotype_mIgG1.fcs	FJComp-PE(yg)-A	Isotype_mIgG1	Isotype_mIgG1
backbone_Plate3_F4_F04_064_target_Isotype_AHIgG.fcs	FJComp-PE(yg)-A	Isotype_AHIgG	Isotype_AHIgG
backbone_Plate3_F11_F11_071_target_Isotype_rIgG2a.fcs	FJComp-PE(yg)-A	Isotype_rIgG2a	Isotype_rIgG2a
backbone_Plate3_F10_F10_070_target_Isotype_rIgG1.fcs	FJComp-PE(yg)-A	Isotype_rIgG1	Isotype_rIgG1

Table 1. Infinity Marker annotation file example.

Z-score Normalization

The original implementation of Infinity Flow uses z-score normalization to "harmonize" the signals of the backbone panel between each of the Infinity Marker captures (Becht, et al., 2021). It is not guaranteed that the distributions observed for each of the backbone markers will be the same between Infinity Marker captures.

One reason this may happen is that the markers in the panel that are not the backbone (the infinity marker or other additional non-backbone marker) may have signals that spill into the backbone channels. For example, PE-conjugated CD48 binds to a very abundant protein on many murine hematopoietic stem and progenitor cells and its signal typically has a much higher mean fluorescence intensity than that of a less abundant antigen, like CD201. The signals across analogous cells from the backbone channels of the CD48 capture could be different from those signals from the CD201 capture because of compensation/unmixing differences caused by the spillover of the PE signal into the backbone channels.

Another reason this could happen is because the captures were done at different times and from separate biological samples. The percentage of cell types will vary across independent biological samples. This can cause changes in the distributions of signals from the backbone channels. For example, if one mouse has slightly more neutrophils than another, the percentage of Ly6G+ cells and the mean fluorescence intensity will be higher in that sample.

Given that the need and benefit of z-score normalization may depend on the experimental conditions used for Infinity Flow, the pyInfinityFlow package makes the normalization step optional, so that the user can optimize the imputation for their own set up.

Artifacts Introduced by Normalization

To assess the necessity of z-score normalization on the accuracy of regression, we generated four new backbone datasets with a 21-color backbone across 4 distinct biological replicates, with 2 samples per biological replicate from >2 million murine bone marrow progenitors. Murine bone marrow was collected from 8-week-old mice on a C57BL/6J genetic background. A Miltenyi Automacs was used to isolate Kit+ hematopoietic stem cells and progenitors. Flow cytometry was performed on two independent 5-laser Cytek Aurora Spectral analyzers. Data was read from the FCS files using the pyInfinityFlow package and XGBoost regression models were trained using half of the backbone markers (11) randomly selected as predictors to predict the other half (10) of the backbone signals on the other biological replicates. We compared the mean squared error of the regression predictions with and without z-score normalization. The average mean squared error increased after z-score normalization ($p<0.001$, paired t-test) (Supplementary Figure 2A), indicating that this normalization strategy can introduce incorrect signal into the predictions. These artifacts are likely due to differences in the sizes of cell type populations between separate biological replicates. Representative flow plots for predictions of Irf8-GFP and Ly6C-BV570 illustrate errors in imputation following z-score normalization (Supplementary Figure 2B).

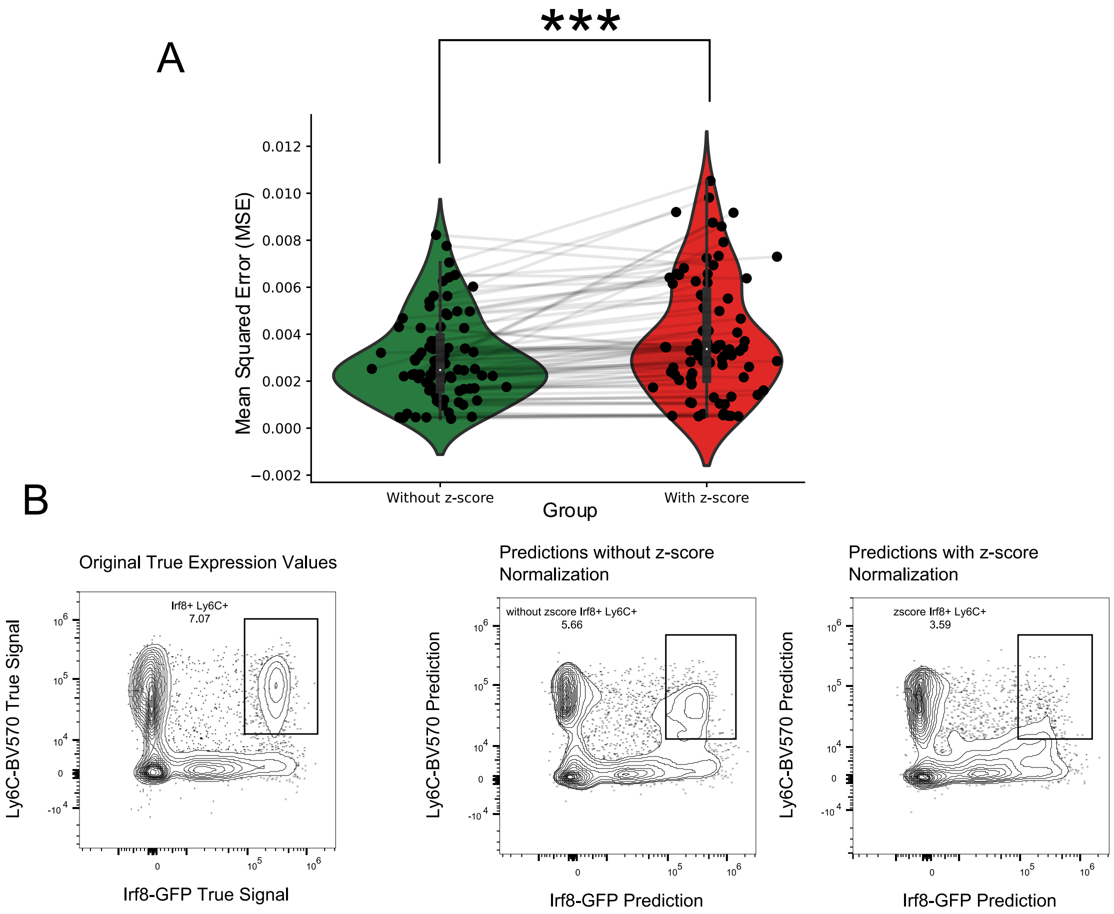

Supplementary Figure 2. **Reduced regression accuracy with z-score normalization of predictors in mouse bone marrow progenitors**. Accuracy assessment of Infinity Marker imputation from independent flow cytometry samples, where the true antibody marker signal is known (Methods). (A) The mean squared error (MSE) of fluorescence channel predictions without (green) and with (red) z-score normalization considering the true antibody signals versus imputed. Each dot represents a fluorescence channel from the backbone that was predicted using an exclusive randomly selected set of backbone channels. The lines indicate how the MSE changed between the condition without z-score normalization to those with z-score normalization. Average MSE increased with z-score normalization ($p < 0.001$, paired t-test). (B) Representative flow plots over markers with poor prediction following z-score normalization. The left plot shows the true signal values, the middle plot shows the predictions without z-score normalization, and the right plot shows predictions with z-score normalization.

Identification of Rare Subpopulations in the Mouse Lung using pyInfinityFlow and cellHarmony

Application of pyInfinityFlow to the same mouse lung dataset produced and analyzed by Becht et al., (2.5 million cells, 253 non-isotype control antibodies) identified 40 Leiden Scanpy defined cell populations. Population labels were inferred from two separate sources: 1) the original labels inferred from Becht et al. and 2) a mouse single-cell RNA-Sequencing (scRNA-Seq) reference cell atlas from LungMAP (Cell Ref 1.0 mouse). Projection of scRNA-Seq labels from corresponding mRNA profiles (antibody to gene names inferred from the NCBI EntrezGene database) was achieved using an embedded call to the cellHarmony algorithm in pyInfinityFlow (DePasquale et al. 2019). The script to perform this is available in the "analysis_scripts" directory of the GitHub repository and a tutorial is provided in the online documentation. For cellHarmony, the scRNA-Seq data was scaled as counts per 10,000 for each gene and for each cell, whereas the infinity markers were normalized with logicle normalization. Despite the differences in technologies used and normalized signals from each, resulting pyInfinityFlow clusters could be divided into prior well-defined lung lineages, including lymphoid (CD4, CD8, NK, ILC, Treg, B-cell, Plasma cell), myeloid (e.g., Alveolar Macrophages, iMon, DC, cDC1, cDC2, Monocytes), epithelial (e.g., AT1, AT2, Mesothelial, Basal, Tuft, Ciliated), endothelial (Cap1, Cap2, VEC, AEC, SVEC) and mesenchymal (fibroblasts) (**Supplementary Figure 3**). Cell-type assignments were considered reliable, as highly related lineages are adjacent to each other in the UMAP plot. These predictions were further broadly similar to those by Becht et al., based on prior reported relative marker abundance for all major cell types reported (**Supplementary Tables 2,3**).

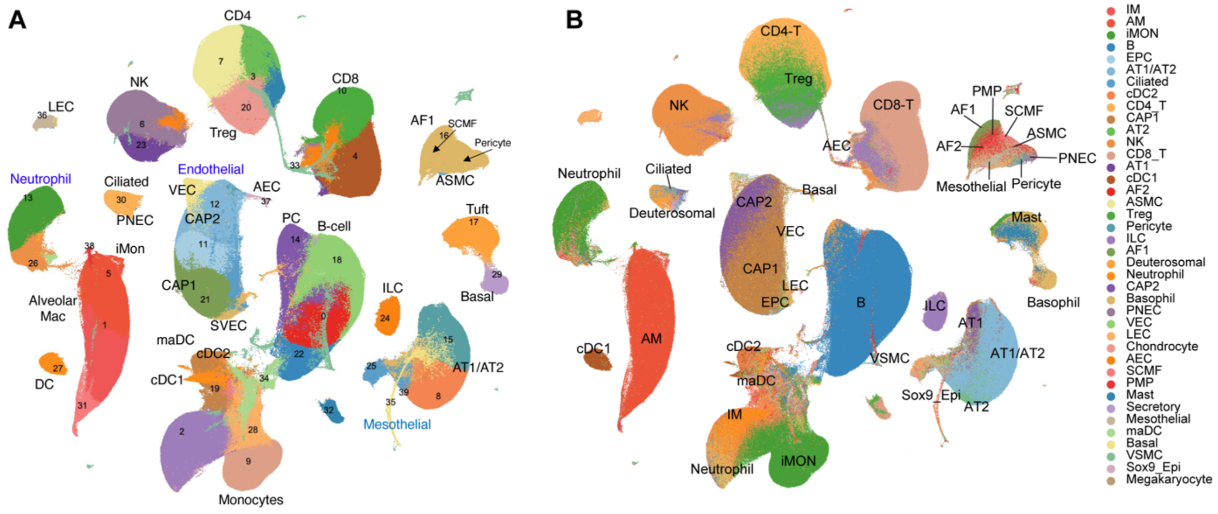

Supplementary Figure 3. **Identification of broad and rare-lung cell populations compared to scRNA-Seq**. A) pyInfinityFlow cluster predictions in 2.5 million mouse lung cells analyzed by Infinity Flow. B) Predicted cell-type annotation labels a large developmental mouse scRNA-Seq dataset produced by LungMAP (Cell Ref 1.0, Guo et al., 2022). Derived labels were produced through centroid-based label projection in the cellHarmony module of pyInfinityFlow from matching antibody associated genes. The cell type labels in panel A are informed from the cell-type predicted labels in panel B and confirmed from Becht et al. 2021.

To determine whether pyInfinityFlow could resolve rare cell types, we further sub-clustering the initial cluster 16 (fibroblasts) to define 11 sub-clusters (**Supplementary Figure 4A,B**). These cell-populations corresponded to highly similar mesenchymal alveolar fibroblasts (AF1, AF2), smooth muscle (VSMC, ASMC), pericytes and secondary crest myofibroblasts (SCMF) based on alignment to mouse scRNA-Seq CellRef 1.0 annotations (Guo, et al., 2022). Here, MarkerFinder nominated differentially expressed surface markers that can be used for the distinction of these cells (**Supplementary Figure 4C** and **Supplementary Tables 3,4**). Simultaneous gating and visualization of the change in UMAP populations allows for the selection of markers to enrich for a given population. pyInfinityFlow will output the 2-dimensional UMAP coordinates as channels in the FCS output file, which can then be analyzed with traditional flow cytometry analysis software (**Supplementary Figure 4D**). Ly-51, CD44, and CD146 were identified as candidate markers for sub-dividing cluster 16. After aligning the gene names that correspond to the detected cell surface proteins in the mouse lung Infinity Flow object (**Supplementary Figure 4D,E** and **Supplementary Tables 3,4**). These markers clearly

delineate mesenchymal subpopulations that correspond to the scRNA-Seq inferred mouse populations.

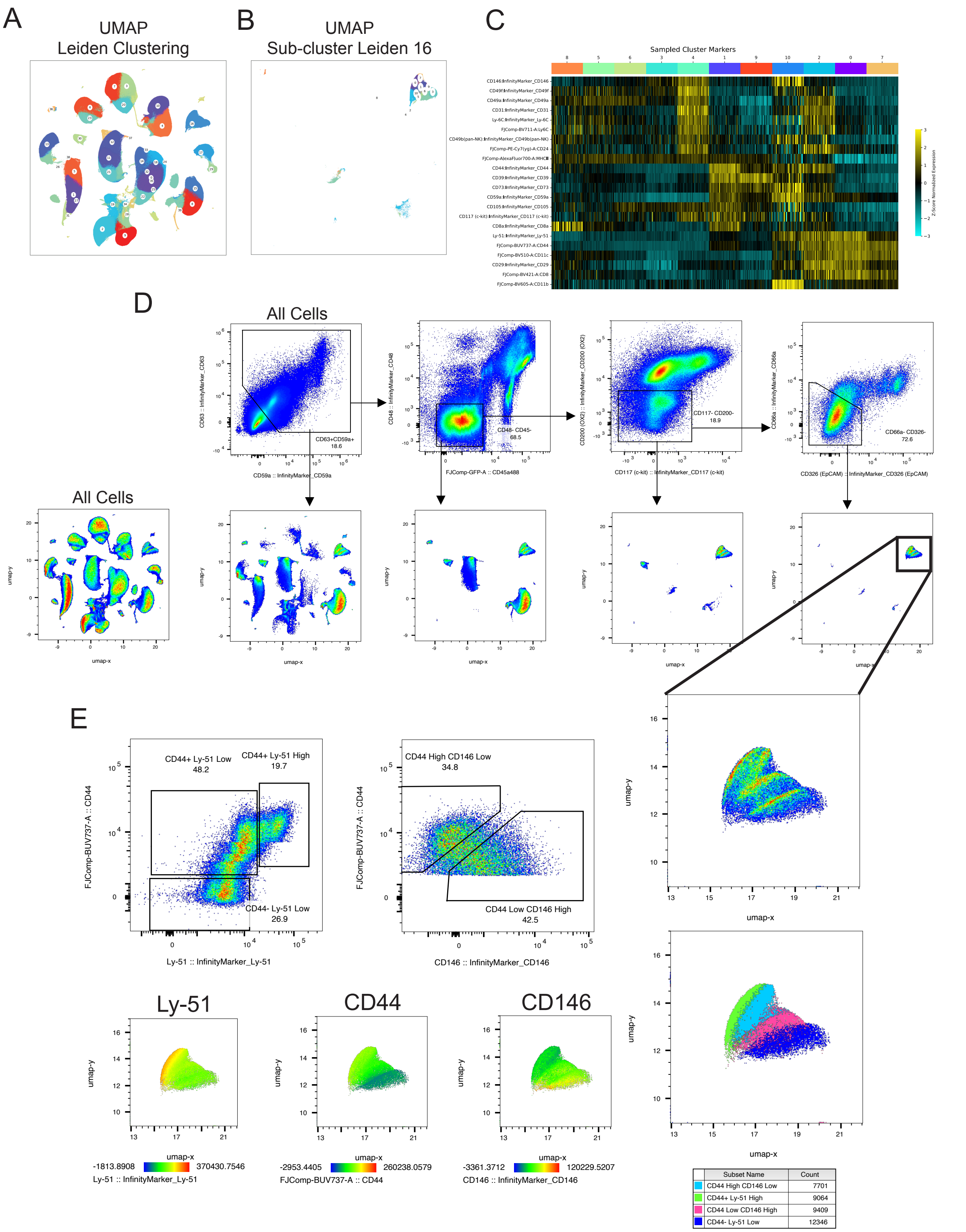

Supplementary Figure 4. Distinct sub-populations and markers of mesenchymal lung cells revealed by pyInfinityFlow. A,B) UMAP representation of all cells after Leiden clustering (A) and subclusters of lung mesenchymal cells in pyInfinity Leiden cluster 16 (B). C) Filtered

markers associated with subclusters of cluster 16. D) Gating scheme to enrich for cluster 16 (upper) and subsequent UMAP positions of cells after gating (lower). E) Gating and visualization of top markers of subclusters of cluster 16.

References

Andersen, M.N., *et al.* Elimination of erroneous results in flow cytometry caused by antibody binding to Fc receptors on human monocytes and macrophages. *Cytometry A* 2016;89(11):1001-1009.

Becht, E., *et al.* High-throughput single-cell quantification of hundreds of proteins using conventional flow cytometry and machine learning. *Sci Adv* 2021;7(39):eabg0505.

Dutertre, C.A., *et al.* Single-Cell Analysis of Human Mononuclear Phagocytes Reveals Subset-Defining Markers and Identifies Circulating Inflammatory Dendritic Cells. *Immunity* 2019;51(3):573-589 e578.

Guo, M., Morley, M.P. and Wu, Y. Guided construction of single cell reference for human and mouse lung *bioRxiv* 2022.

Moore, W.A. and Parks, D.R. Update for the logicle data scale including operational code implementations. *Cytometry A* 2012;81(4):273-277.

Venkatasubramanian, M., *et al.* Resolving single-cell heterogeneity from hundreds of thousands of cells through sequential hybrid clustering and NMF. *Bioinformatics* 2020;36(12):3773-3780.
